# Mechanical behavior of hybrid custom implant abutments with various crown materials: a 3D finite element analysis

**DOI:** 10.1186/s12903-025-06445-w

**Published:** 2025-07-05

**Authors:** Pongsakorn Poovarodom, Guilherme Faria Moura, Fabio Antonio Piola Rizzante, Chaiy Rungsiyakull, Jarupol Suriyawanakul, Pimduen Rungsiyakull

**Affiliations:** 1 Department of Reconstructive and Rehabilitation Sciences, James B. Edwards College of Dental Medicine, Medical University of South Carolina, Charleston, USA; 2 Department of Mechanical Engineering, Faculty of Engineering, Chiang Mai University, Chiang Mai, Thailand; 3 Department of Mechanical Engineering, Faculty of Engineering, Khon Kaen University, Khon Kaen, Thailand; 4 Department of Prosthodontics, Faculty of Dentistry, Chiang Mai University, Chiang Mai, Thailand

**Keywords:** Customized abutments, CAD/CAM, Material properties, Finite element analysis, Mechanical performance

## Abstract

**Background:**

Traditional and innovative materials are widely used in dentistry; however, the mechanical behavior of hybrid custom implant abutments, particularly stress distribution in various material combinations, is not fully understood. The study aims to evaluate the mechanical behavior of hybrid custom implant abutments made from various material combinations, including their effects on von Mises stress, maximum and minimum principal stresses, and deformation.

**Methods:**

Two 3-dimensional (3D) models were constructed: a 1.5 mm subcrestal as the test and an equicrestal model as the control. The subcrestal model explored seven materials (Zirconia, Titanium, Lithium Disilicate, Polymer-Infiltrated Ceramic Networks, PEEK, PEEK reinforced with carbon fiber and reinforced with glass fiber) in various abutment and crown combinations. Each model included an implant, titanium base abutment, abutment screw, a custom abutment, a zirconia crown, and bone. A 200 N load was applied, and a Finite Element Analysis (FEA) assessed peak, volume average, and distribution of von Mises stress and principal stress.

**Results:**

The titanium base (Tibase) exhibited the highest peak and volume average von Mises stresses (306–429 MPa), followed by the custom abutment (40–95 MPa) and crown (46–81 MPa). Material changes significantly impacted stress distribution in the Tibase and customized abutments. PICN, Zirconia, Titanium, and Lithium Disilicate abutments showed peak principal stresses between 77 and 85 MPa, while PEEK variants reduced stress in the custom abutment (35–66 MPa) but increased it in the Ti-base (356–405 MPa). PEEK also increased minimum principal stresses in the Ti-base (-400 to -600 MPa).

**Conclusions:**

Abutment materials have a greater impact on stress outcomes compared to crown materials. Abutments with high Young’s modulus contribute to increased core system stiffness in hybrid custom abutment complexes. Choosing abutment materials with a high Young’s modulus for hybrid custom implant abutments is essential to optimize stress distribution and enhance the stability of the implant system.

## Background

The integration of Computer-Aided Design and Computer-Aided Manufacturing (CAD/CAM) technologies in implant dentistry has revolutionized the field by offering unprecedented precision and customization in the creation of dental restorations [1]. Among these developments, hybrid custom implant abutments — combining different materials such as titanium, zirconia, lithium disilicate, and polyetheretherketone (PEEK) — have gained popularity due to their potential to optimize both esthetics and biomechanics [2–4].

Different restorative materials influence the mechanical behavior of implant-abutment-crown complexes, which in turn affects load transmission to the surrounding bone and implant components [5–7]. Titanium and zirconia, with high elastic moduli, are widely accepted for their strength and biocompatibility, while materials like lithium disilicate and PICN (polymer-infiltrated ceramic network) offer an improved balance between esthetics and functional durability [8, 9]. More recently, PEEK and its composites have been proposed as promising alternatives for abutments and frameworks due to their flexibility, low weight, and favorable stress-shielding effects [10–12].

However, the use of multiple materials in a single restorative complex introduces a range of mechanical interactions that are not yet fully understood. In particular, hybrid abutment designs pose challenges in stress distribution across the crown, abutment, and implant interface. These stress variations may contribute to mechanical complications, such as screw loosening, abutment fracture, or bone resorption [13, 14].

Finite Element Analysis (FEA) has proven to be a valuable tool in exploring the mechanical implications of dental implant systems in silico, offering insights into stress distribution under static and dynamic conditions [15–17]. Several studies have employed 3D FEA to examine factors such as material combinations [13], crown stiffness [18], prosthetic screw configurations [19], and the mechanical performance of different adhesive materials in indirect restorations [20, 21]. These investigations collectively highlight the importance of material selection in implant-supported prostheses and establish FEA as a foundational methodology for optimizing restorative strategies.

Despite the growing body of literature, there is limited research systematically comparing the mechanical behavior of hybrid custom abutments incorporating both current and advanced materials under clinically relevant loading conditions. Additionally, previous studies often focus on individual components rather than the entire implant-abutment-crown system. Therefore, a comprehensive mechanical analysis of various material pairings is necessary to guide clinicians in selecting appropriate combinations for long-term success.

The aim of this study is to evaluate the mechanical behavior of hybrid custom implant abutment complexes fabricated with different combinations of abutment and crown materials using 3D nonlinear finite element analysis. The null hypothesis is that different material combinations do not significantly affect stress distribution patterns or magnitudes within the hybrid implant system.

## Methods

**Fig. 1:**
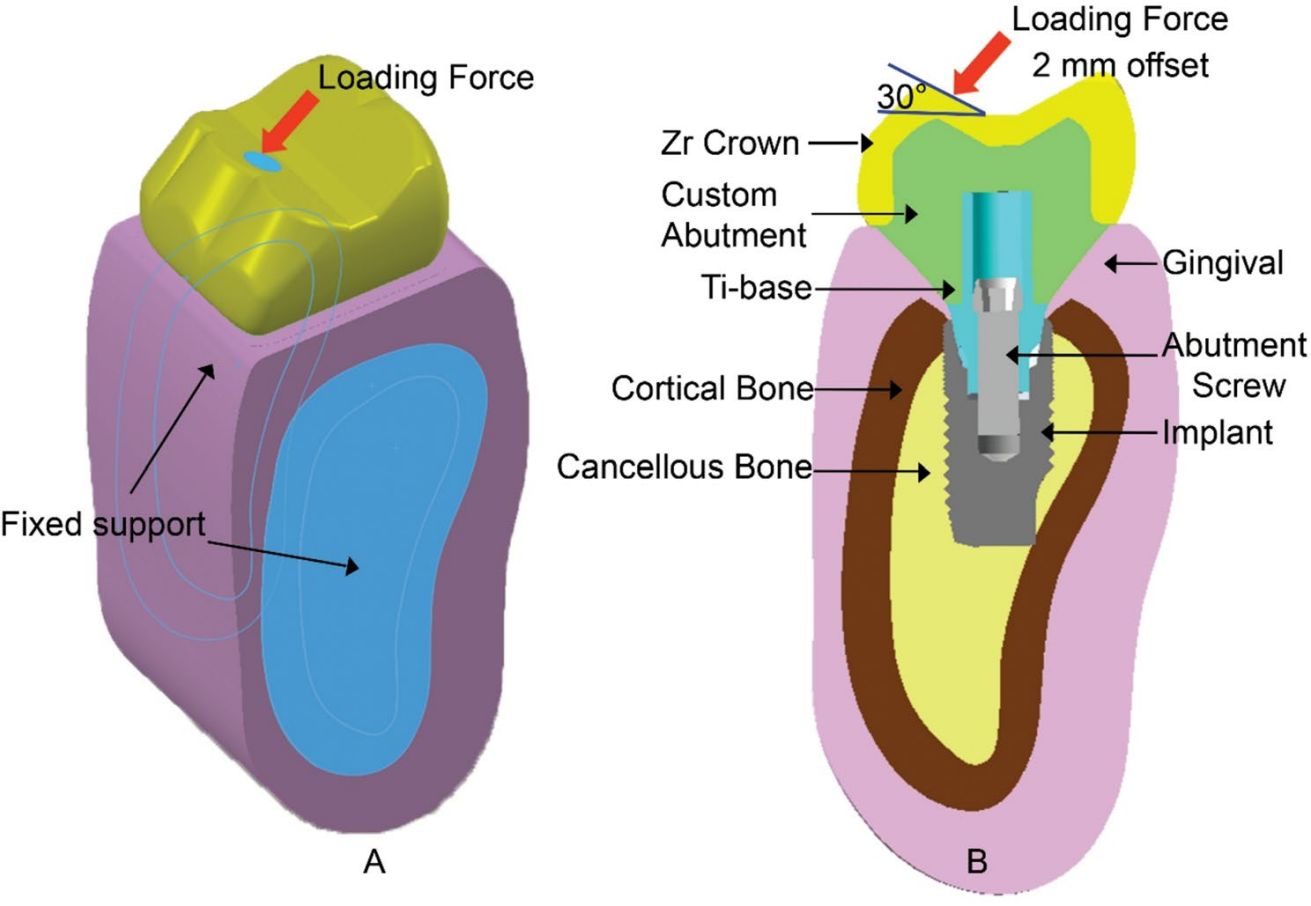
**A**, Full isotropic view of finite element model with loading and boundary condition. **B**, bucco-lingual cross section view of testing model with components label

**Fig. 2:**
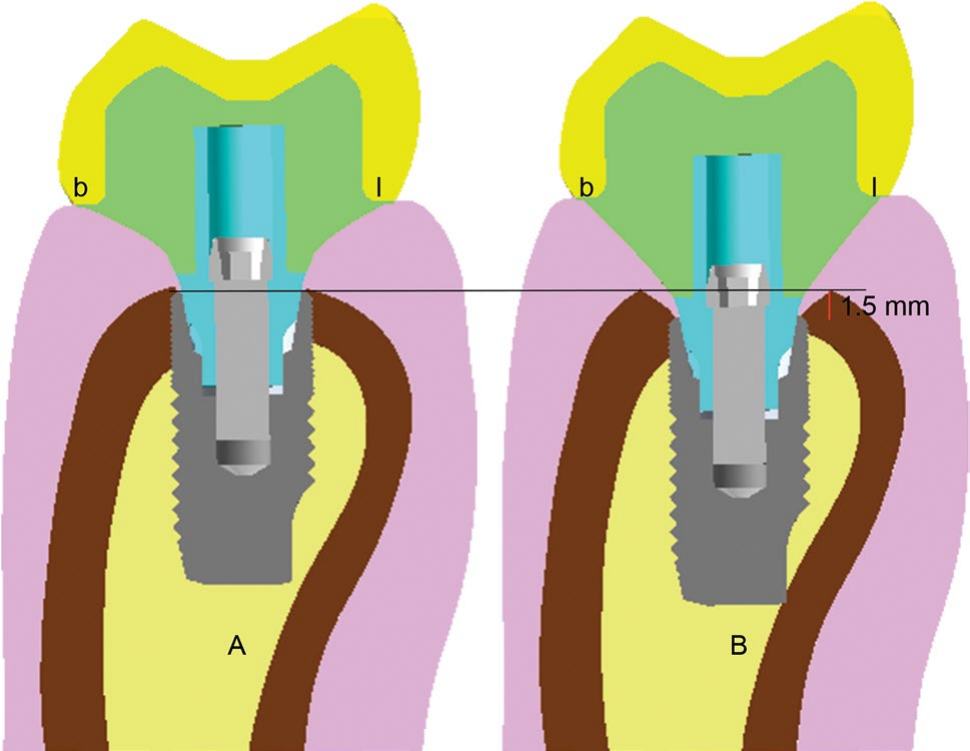
Bucco-lingual cross section views of **A**, Control model and **B**, Subcrestal model. **b**, buccal side; l, lingual side

Two 3-dimensional (3D) finite element models were constructed: a control model with an implant at the crestal bone level and a test model with a 1.5 mm subcrestal implant placement [9, 12], referred to as the subcrestal position (Figs. 1 and 2). In this study, a 1.5 mm subcrestal placement model was used to explore seven different materials for various combinations of abutment and crown materials. These materials included Zirconia (Zir), Titanium (Ti), Lithium Disilicate (LDS), Polymer-Infiltrated Ceramic Networks (PICN), Polyetheretherketone (PEEK), PEEK reinforced with carbon fiber (PEEK-C), and PEEK reinforced with glass fiber (PEEK-G). This approach facilitates the analysis of forty-three different material combinations for the crown/abutment implant complex. Additionally, the study compared these combinations to two control models placed at the equal crestal position: one with a customized titanium abutment and zirconia crown, and the other with a customized zirconia abutment on a titanium base (Tibase) paired with a zirconia crown. These controls reflect common clinical practice. The modeling process followed a CAD/CAM-inspired digital workflow to replicate clinically relevant component geometries prior to finite element analysis.

**Fig. 3:**
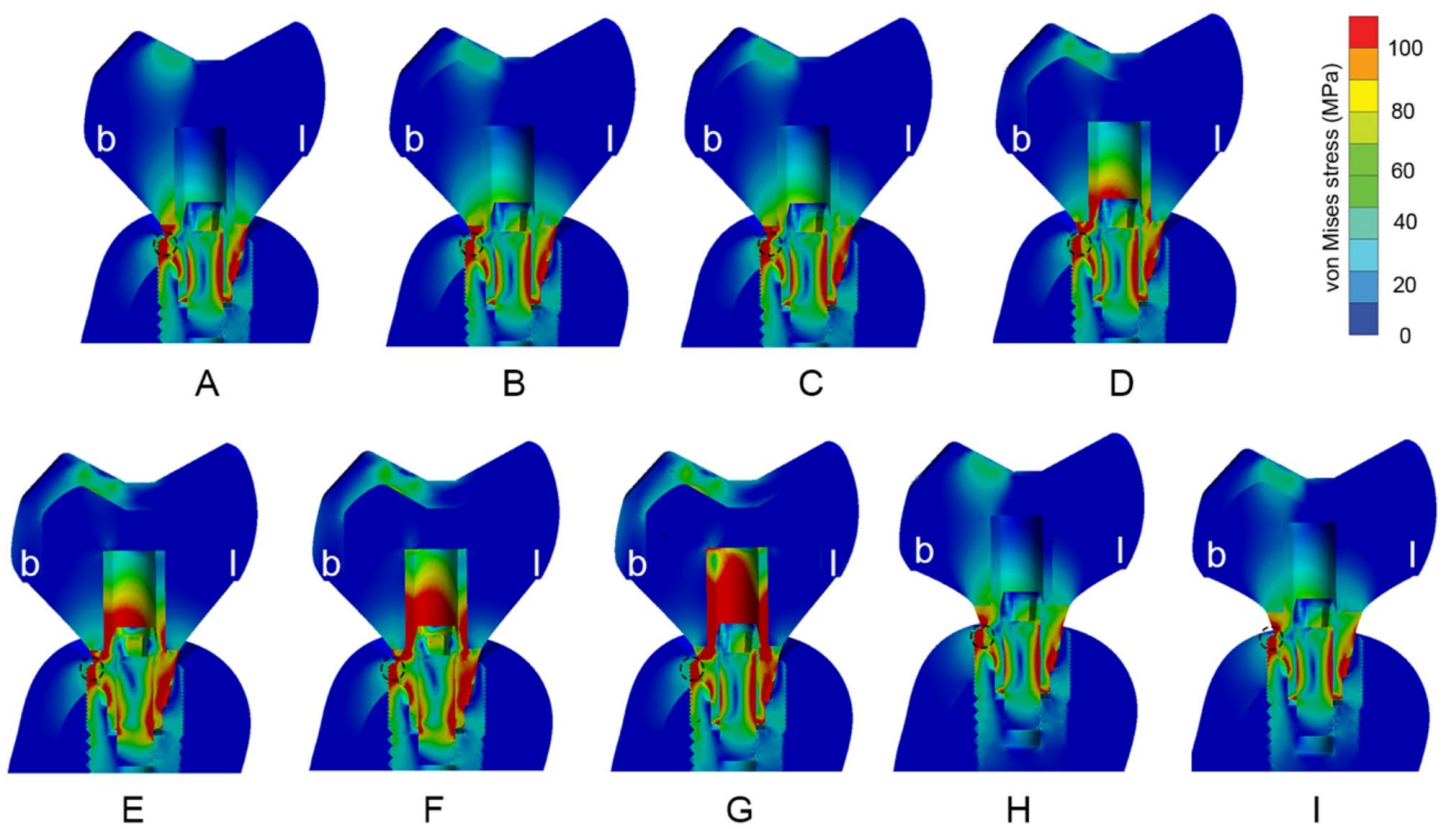
Buccolingual cross-sectional view of all tested models of von Mises stress distribution in **A**, Zirconia abutment. **B**, Titanium abutment.**C**, Lithium disilicate abutment. **D**, PICN abutment. **E**, PEEK-C abutment.**F**, PEEK-G abutment. **G**, PEEK abutment. **H**, Control zirconia abutment. **I**, Control titanium abutment. Black circles represent the location of maximum von Mises stress in each model. b, buccal side; l, lingual side

**Fig. 4:**
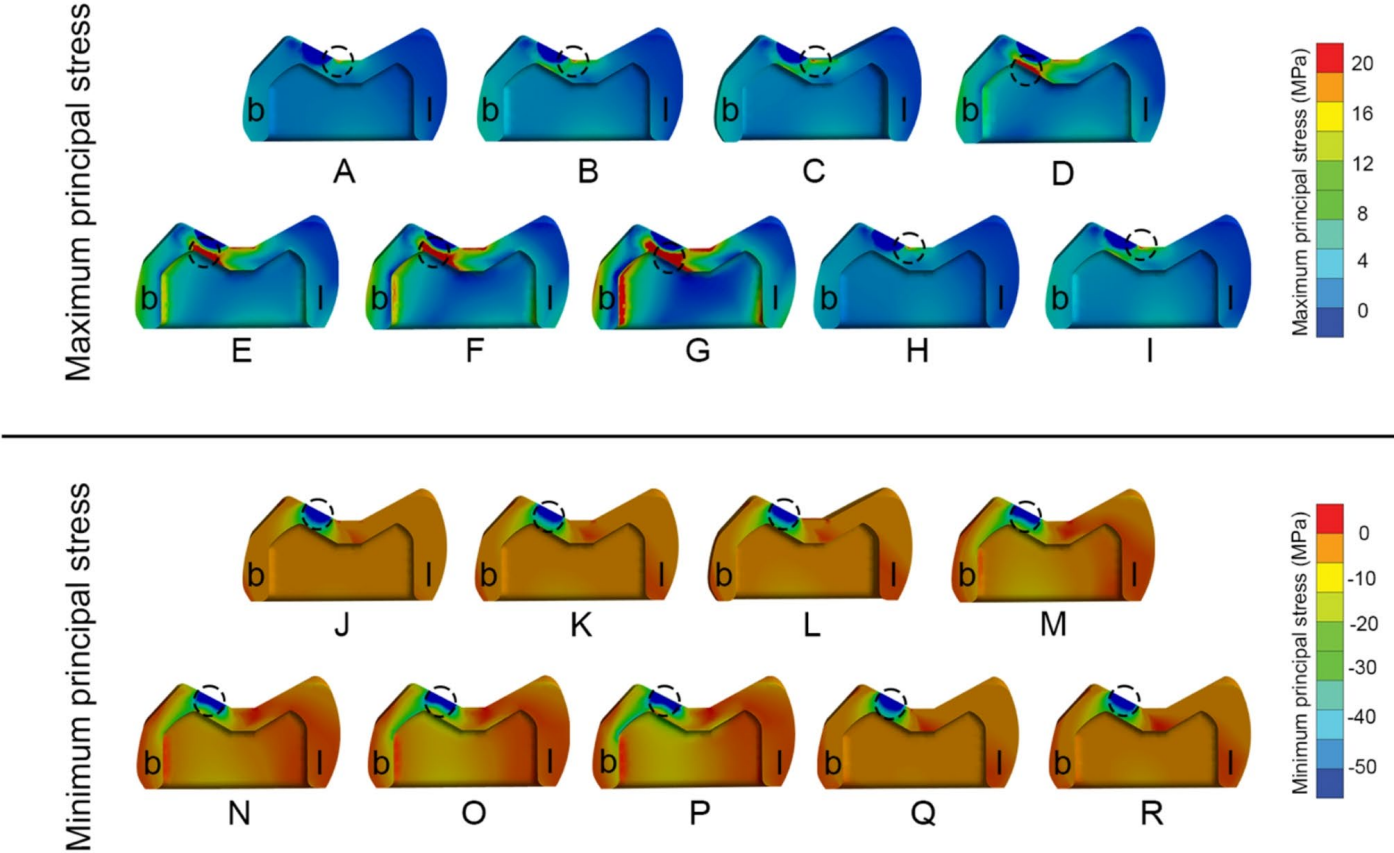
Buccolingual cross-sectional view of all crown models. **(b)** buccal side, **(l)** lingual side, of maximum (upper row) and minimum principal stress (lower row) distribution in: **(A, J)** Zirconia abutment. **(B, K)** Titanium abutment. **(C, L) **Lithium disilicate abutment. (D, M) PICN abutment. (E, N) PEEK-C abutment. (F, O) PEEK-G abutment. (G, P) PEEK abutment. (H, Q) Control zirconia abutment. (I, R) Control titanium abutment. Black circle represents location of peak (in magnitude) maximum and minimum principal stress

**Fig. 5:**
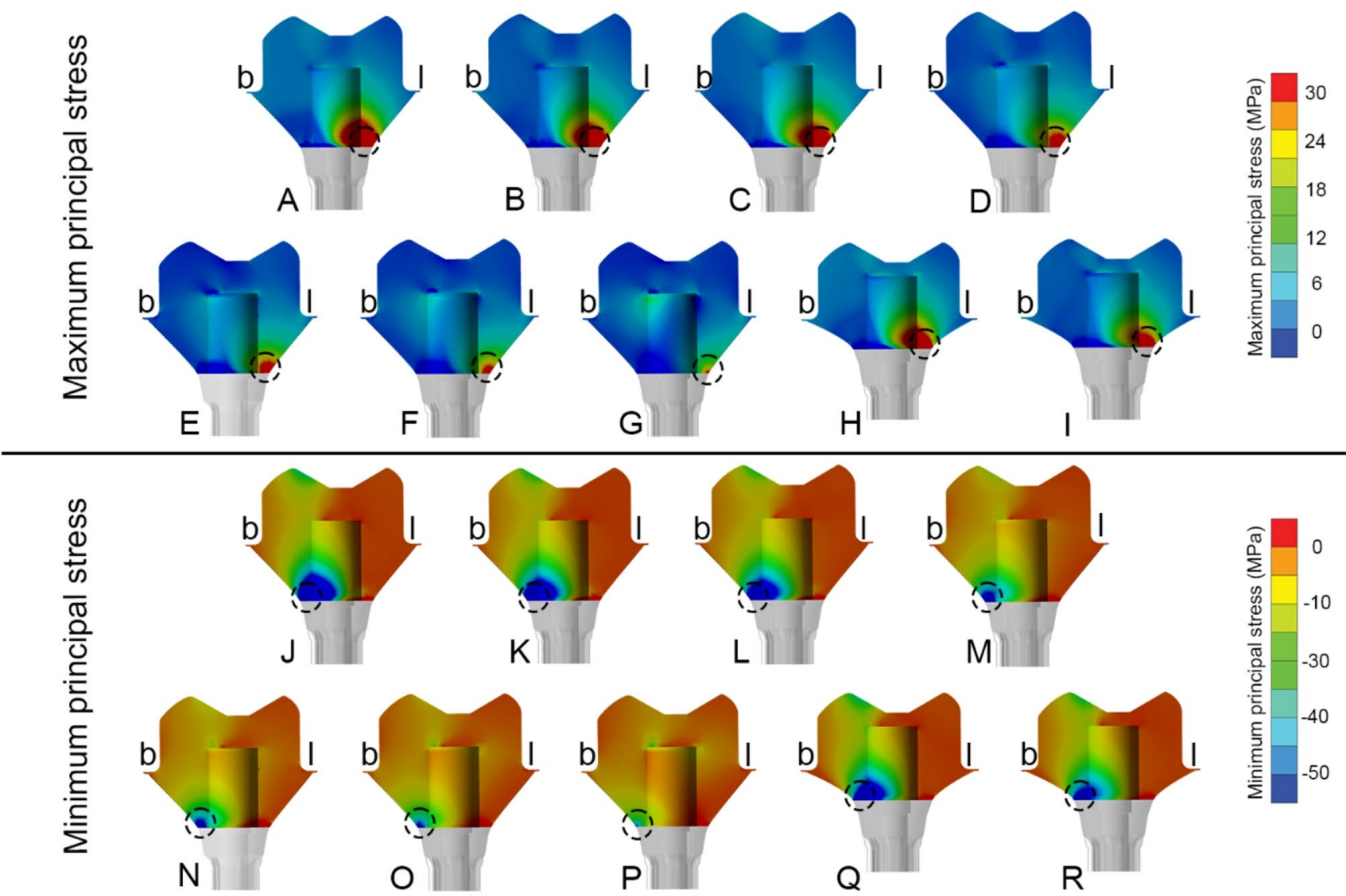
Buccolingual cross-sectional view of all customized abutment models. **(b)** buccal side, **(l)** lingual side, of maximum (upper row) and minimum principal stress (lower row) distribution in: (A, J) Zirconia abutment. (B, K) Titanium abutment. (C, L) Lithium disilicate abutment. (D, M) PICN abutment. (E, N) PEEK-C abutment. (F, O) PEEK-G abutment. (G, P) PEEK abutment. (H, Q) Control zirconia abutment. (I, R) Control titanium abutment. Black circle represents location of peak (in magnitude) maximum and minimum principal stress

**Fig. 6:**
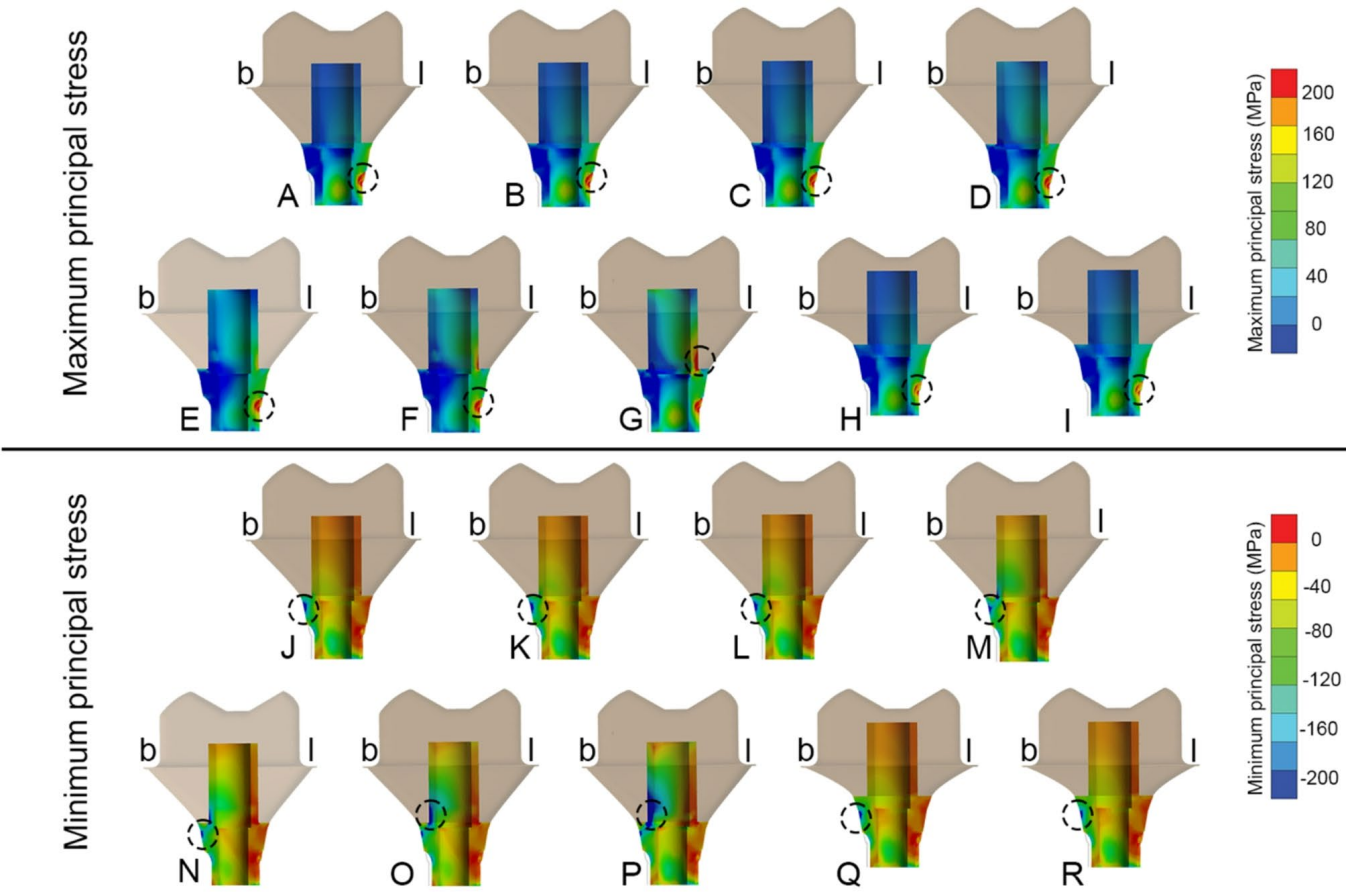
Buccolingual cross-sectional view of all titanium base abutment (Tibase) models. (b) buccal side, (l) lingual side, of maximum (upper row) and minimum principal stress (lower row) distribution in: (A, J) Zirconia abutment. (B, K) Titanium abutment. (C, L) Lithium disilicate abutment. (D, M) PICN abutment. (E, N) PEEK-C abutment. (F, O) PEEK-G abutment. (G, P) PEEK abutment. (H, Q) Control zirconia abutment. (I, R) Control titanium abutment. Black circle represents location of peak (in magnitude) maximum and minimum principal stress

To simulate anatomical structures, a section of mandibular bone from the posterior molar region was obtained using cone beam computed tomography (CBCT) imaging (Varaview X800; MORITA). The DICOM data were segmented and converted into 3D geometry using Rhinoceros 3D (Robert McNeel and Associates), enabling accurate modeling of the cortical and cancellous bone layers. The cortical bone was represented as a 2 mm outer shell, while the cancellous core was modeled as a bonded internal volume [22].

Implant components were designed based on manufacturer specifications. A virtual Ø5.4 × 11-mm dental implant (OsseoSpeed EV, AstraTech; Dentsply Sirona) was placed into the bone model. A titanium base abutment (AT EV 5.4 GH1 L, Dentsply Sirona) with a retention height of 5 mm and 1 mm gingival height was modeled along with a Ø2 × 7.6-mm abutment screw (Screw AT EV 5.4, Dentsply Sirona). The custom abutment and crown were designed using SolidWorks (SolidWorks 2017; Dassault Systèmes), simulating clinically relevant geometries. The crown measured 10 mm buccolingually, 12 mm mesiodistally, and had an interocclusal space of 8 mm.

CAD models of all components—including bone, implant, Tibase, screw, abutment, and crown—were exported in STEP format and imported into the ANSYS Workbench 2020 environment for preprocessing and simulation. Prior to meshing, interfaces were aligned to ensure accurate contact behavior. Mesh convergence analysis was performed to determine optimal element sizes, ensuring both simulation accuracy and computational efficiency.

This structured workflow—from CBCT-based anatomical modeling to CAD component design and FEA preprocessing—was intended to closely replicate the digital planning and manufacturing steps used in contemporary implant prosthodontics.

For the boundary conditions, the contact between the implant-abutment and abutment-screw was treated as frictional contact, with a friction coefficient set to 0.4 [23]. Additionally, the contact at the interface between the abutment screw thread surface and the internal thread of the implant was modeled using a single right-handed thread type, featuring a mean pitch diameter of 1.88 mm, a pitch distance of 0.4 mm, and a thread angle of 37.1 degrees.

The abutment screw was tightened to 25 Ncm, as specified by the Bolt Pretension function. Unlike the abutment screw connection, a bonded contact was utilized for the interfaces between the bone and the implant, as well as between the abutment and the crown. The titanium base abutment, zirconia custom abutment, and zirconia crown were modeled to be fully seated without any gaps, excluding the cement layer to simplify the modeling process.

Table 1 provides a detailed listing of the mechanical properties for the implant, abutment, screw, and crown. To streamline the analysis, all materials were modeled as isotropic, homogeneous, and linearly elastic. Similarly, the mechanical properties of the surrounding hard tissues were also assumed to be linearly elastic and isotropic, allowing for a simplified and consistent approach to the analysis [24].

**Table 1:** Material properties of implant fixture, implant abutment and crown materials

Materials/ properties	Young’s modulus (GPa)	Poisson’s ratio	Yield strength (MPa)
Titanium CP grade IV [25]	110	0.3	480
Zirconia (Zr) [26]	200	0.35	900
Lithium disilicate (LDS) [26]	95	0.3	360
PICN [27]	30	0.3	160
PEEK-C [28]	19.7	0.42	140
PEEK-G [28]	10.5	0.35	140
PEEK [28]	3.35	0.36	140
Cortical bone [29]	11.67	0.3	100
Cancellous bone [29]	1.872	0.3	20

A static load of 200 N [30] was applied to the occlusal surface, offset 2 mm horizontally toward the buccal side and inclined at a 30-degree angle from the vertical axis. The boundary conditions for fixed support were assigned to the mesial and distal sectional surfaces of the bone model to simulate surrounding bone support. The finite element (FE) mesh densities for all components in this study were validated through a convergence study, ensuring both accuracy and computational efficiency. The convergence study determined that an element size of 0.4 mm was optimal. At the contact surface between the abutment screw and the implant, a contact size of 0.1 mm was specified.

A 3D 10-node tetrahedral solid element (SOLID187) was utilized, with each of the four finite element models comprising an average of 900,000 elements and 600,000 nodes. The finite element analysis (FEA) was conducted using ANSYS Workbench 2020 software (Swanson Analysis Inc) on workstation computer (13th Gen Core (TM) i9-13900 K CPU 24 Core 3.0 GHz).

The study investigated the effects of various restorative materials used in hybrid customized abutments and crowns. It focused on five key parameters: peak von Mises stress, volume average von Mises stress, von Mises stress distribution, and peak (in magnitude) of maximum and minimum principal stress. Von Mises stress was used to assess the overall mechanical performance and yielding potential of ductile materials (e.g., titanium, PEEK, PICN). It provides a scalar value representing the combined effect of multidirectional stresses and is widely used in dental FEA to predict material failure under complex loading conditions. Maximum principal stress was used to evaluate regions under tensile load, which is critical for assessing the fracture potential of brittle materials such as zirconia and lithium disilicate. Conversely, minimum principal stress (representing compressive stress) was analyzed to evaluate compressive loads, especially relevant to understanding how stiff materials influence stress absorption and transfer.

To address potential biases from peak stress artifacts in the models, the concept of volume-averaged von Mises stress was employed. This method integrates and averages stress values over a specific region of each element, taking into account both the volume of individual elements and the total volume of all elements, as defined by Eq. 1. By using volume average von Mises stress, the study assessed the average stress experienced by each element across all models. This approach facilitated a meaningful comparison between different material combinations, helping to identify which component experienced the highest overall stress in each model. This comprehensive analysis allowed for the evaluation of how different material combinations of abutment and crown influence stress distribution in the hybrid customized abutment complex, providing insights into their mechanical performance under various conditions [31].

1 $$\text{Volume average von Mises stress} = \frac{\sum (\text{stress} \times \text{volume by element})}{\sum \text{volume}}$$.

## Results

The von Mises stress analysis demonstrated consistent stress distribution patterns across all models (Fig. 3). Stress concentrations were primarily localized at the anti-rotational area of the Tibase on the lingual side, the micro threads of the implant on the buccal side, and the abutment-screw interface. Notably, abutments made from materials with lower Young’s modulus such as PEEK and its variants produced higher stress concentrations at the Tibase’s retentive post (Fig. 3E–G). The highest stress occurred at the Morse taper connection at the implant platform.

Among all components, the Tibase exhibited the highest peak von Mises stress values, ranging from 306 to 429 MPa (Table 2). Customized abutments showed moderate stress levels (40–95 MPa), while crowns experienced the lowest stresses (46–81 MPa). This pattern was consistent in volume-averaged von Mises stress results, with the Tibase showing the greatest accumulation (55–99 MPa), followed by the abutment (3–9 MPa), and the crown (0.7–6 MPa) (Tables 2, 3 and 4).

**Table 2:**
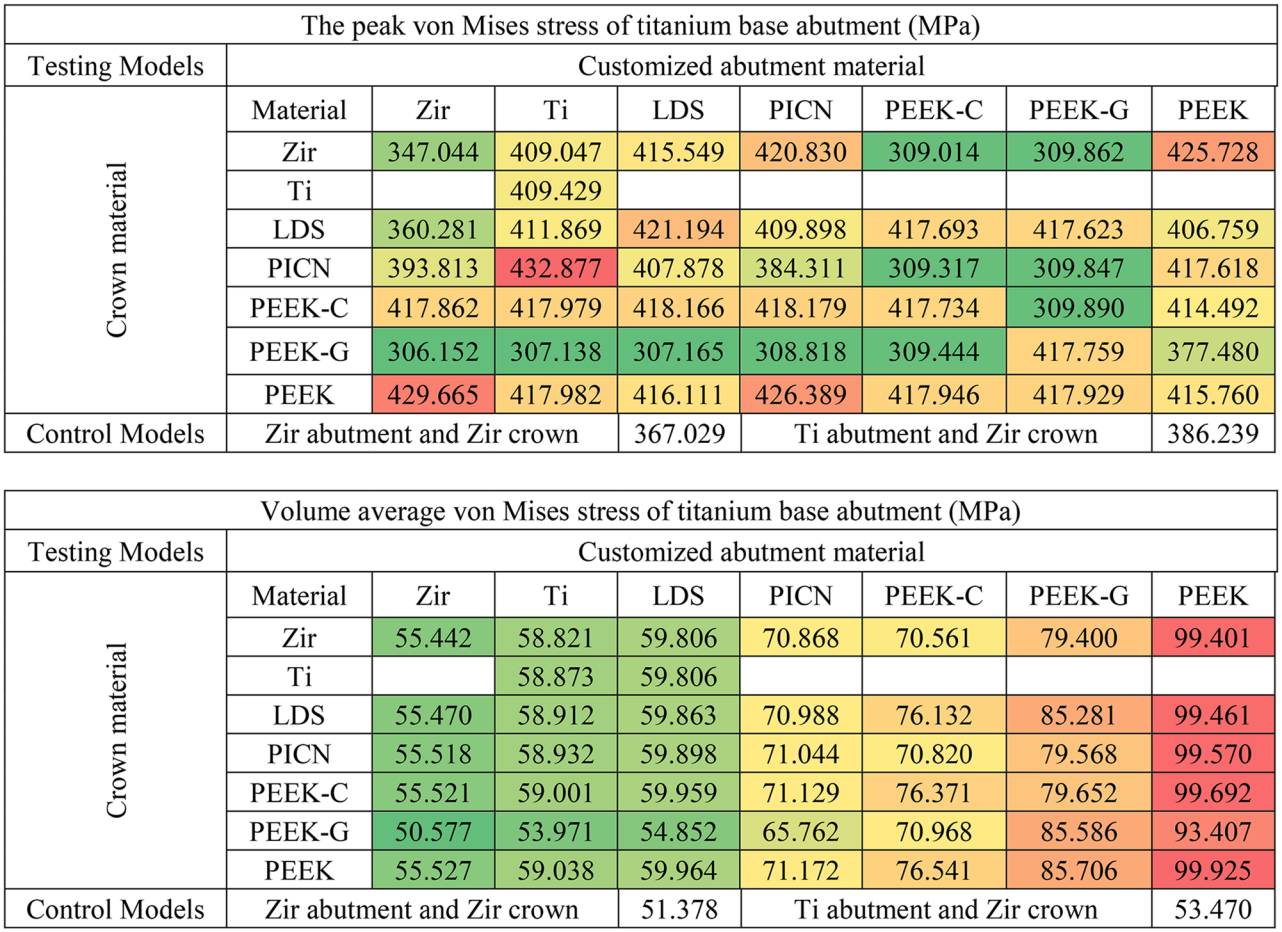
Peak and volume average of von Mises stress of titanium base abutment in each model (red, orange, yellow, and green indicate stress values on a conditional color scale, which ranges from the upper to lower bound)

**Table 3:**
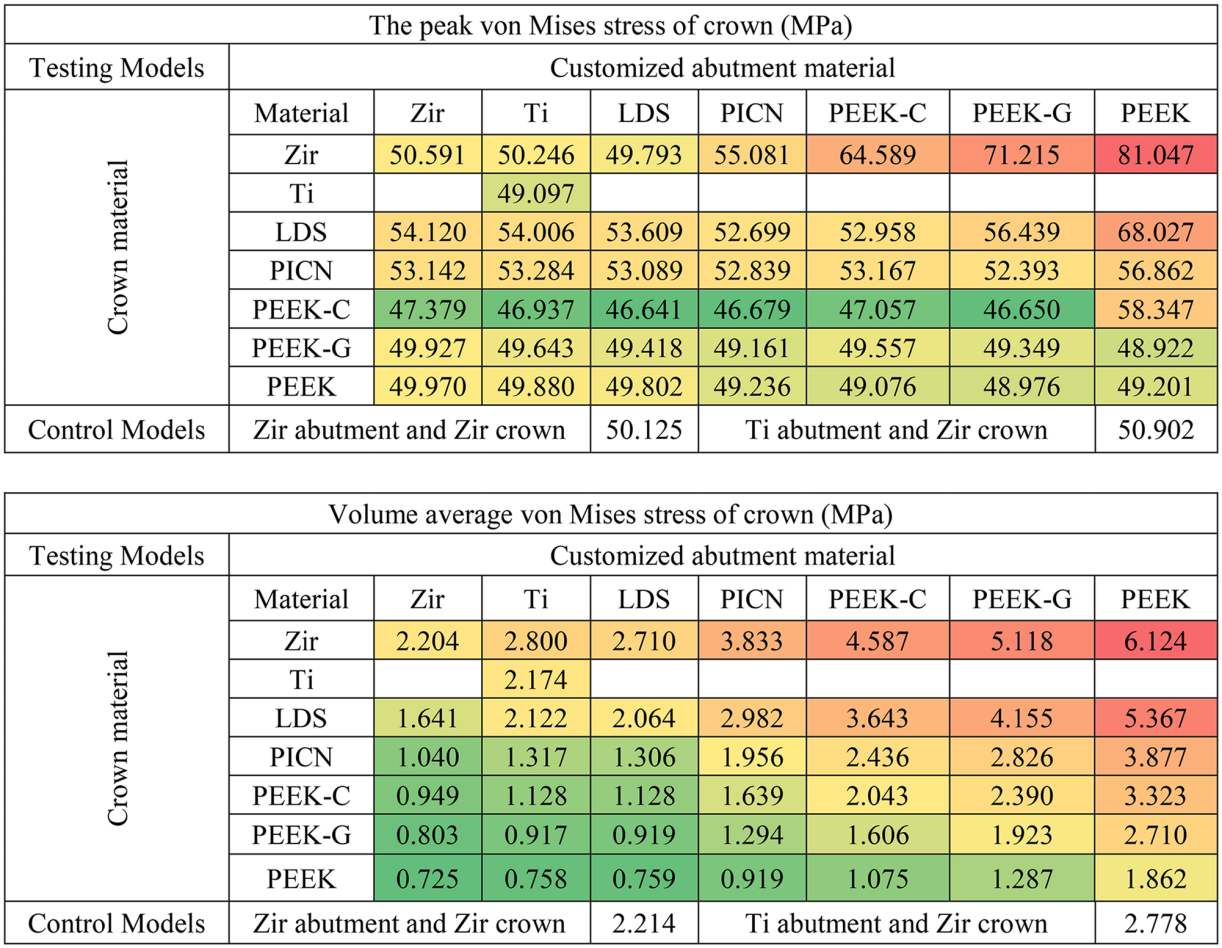
Peak and volume average of von Mises stress of crown in each model (red, orange, yellow, and green indicate stress values on a conditional color scale, which ranges from the upper to lower bound)

**Table 4:**
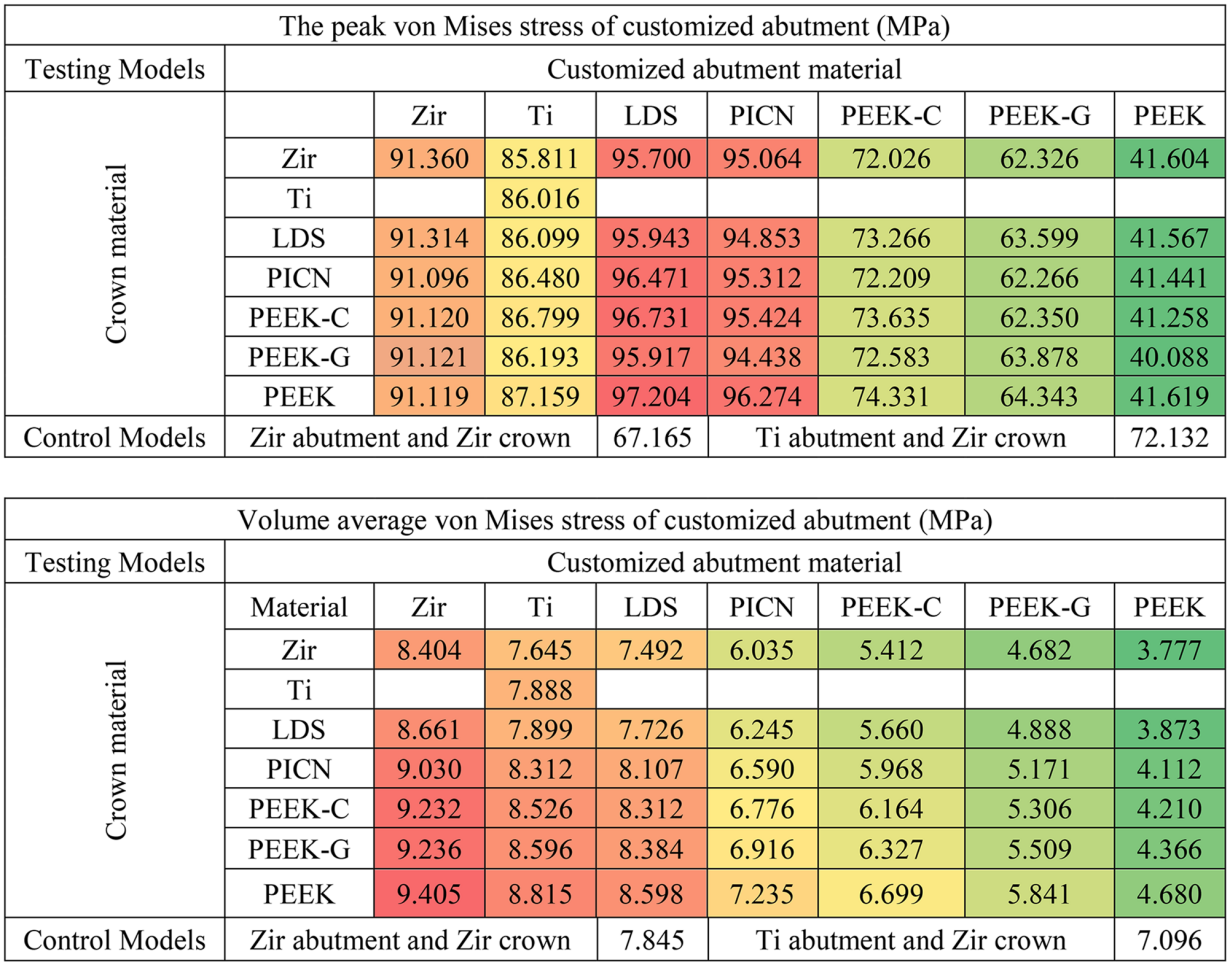
Peak of maximum and minimum principal stress of customized abutment in each model (red, orange, yellow, and green indicate stress values on a conditional color scale, which ranges from the upper to lower bound)

Principal stress analysis further highlighted these trends. In the crown, peak maximum and minimum principal stresses were concentrated in the occlusal loading area (Fig. 4). Crowns supported by PEEK abutments showed the largest regions of high principal stresses, while zirconia-supported crowns showed lower stress fields (Fig. 4A vs. 4G). In the customized abutments, materials with higher stiffness (PICN, Zir, Ti, LDS) yielded peak maximum principal stresses between 77 and 85 MPa and minimum stresses between − 120 and − 128 MPa (Table 5). PEEK abutments demonstrated reduced stress within the abutment but transferred greater loads to the Tibase.

**Table 5:**
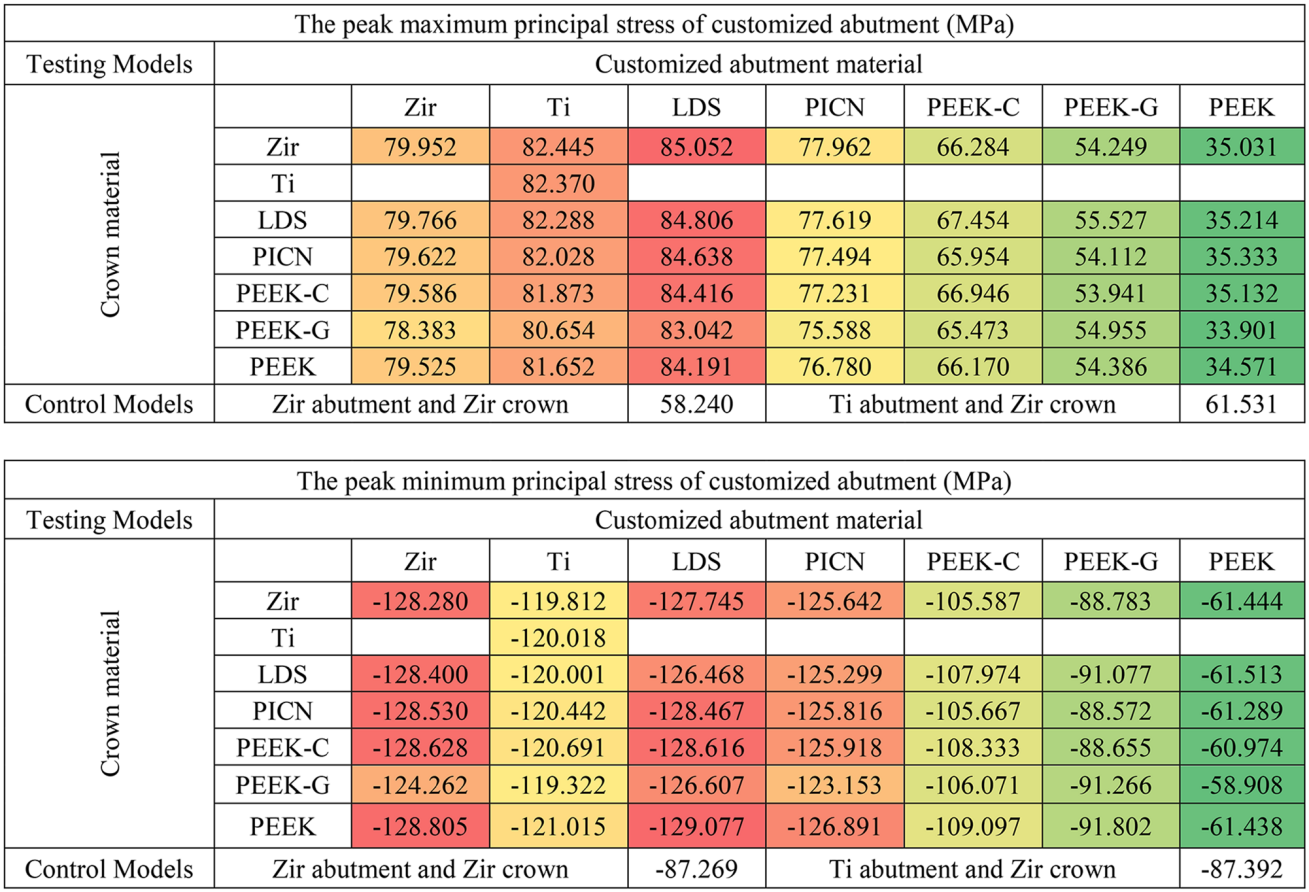
Peak of maximum and minimum principal stress of customized abutment in each model (red, orange, yellow, and green indicate stress values on a conditional color scale, which ranges from the upper to lower bound)

This trend reversed in the Tibase, where PEEK-based models showed markedly elevated principal stresses. Maximum principal stress in these models reached 405 MPa, compared to 305–330 MPa in stiffer material groups. Similarly, minimum principal stresses reached as low as -600 MPa in PEEK models, versus − 300 MPa in others (Table 6). These results indicate a shift in stress concentration from the abutment to the Tibase when using lower-modulus materials.

**Table 6:**
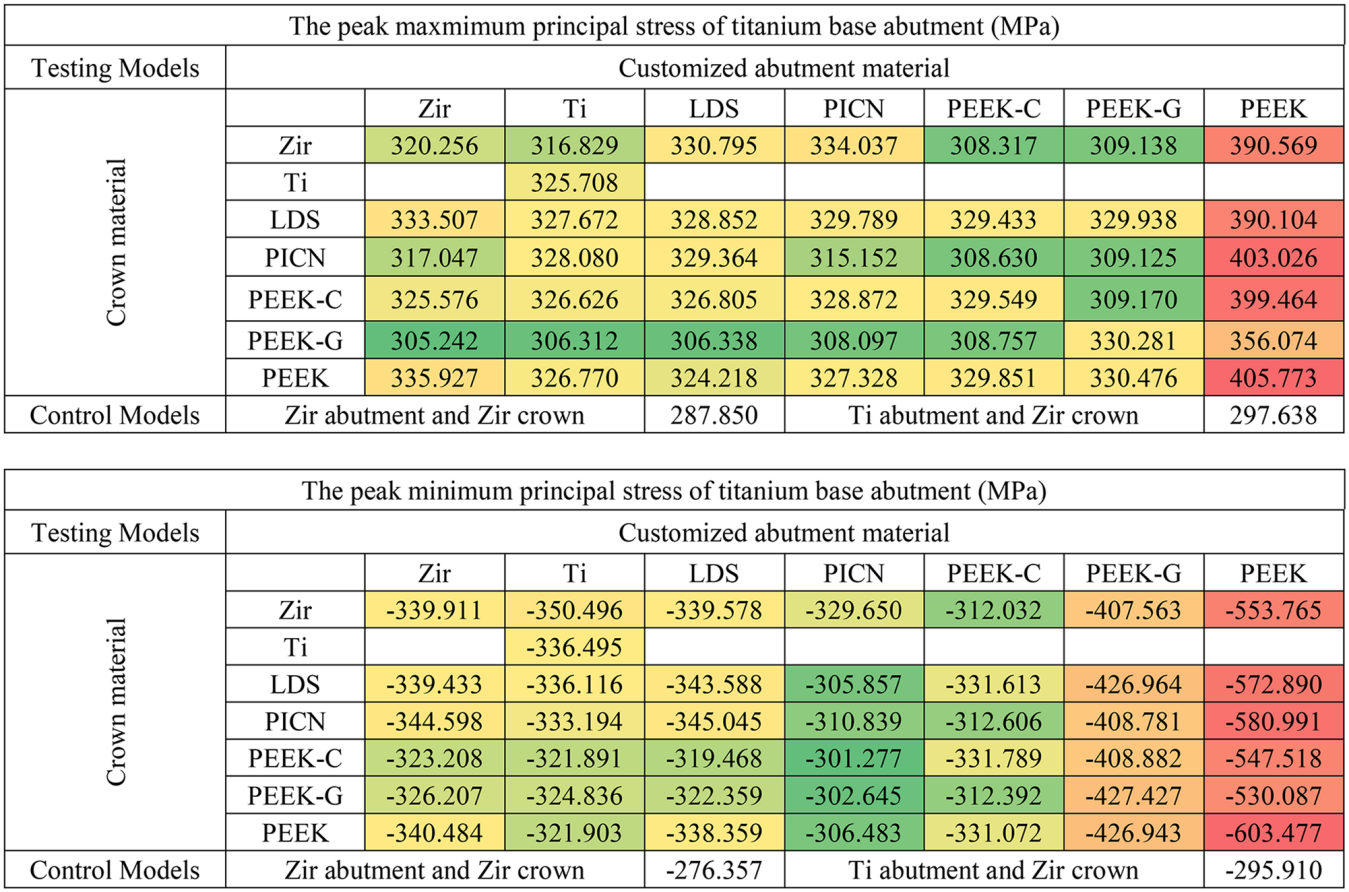
Peak of maximum and minimum principal stress of titanium base abutment in each model (red, orange, yellow, and green indicate stress values on a conditional color scale, which ranges from the upper to lower bound)

## Discussion

This investigation demonstrated that the mechanical response in a hybrid customized abutment complex is significantly affected by the materials chosen for the crown and customized abutment, especially regarding stress concentration. These findings highlight the importance of selecting appropriate customized abutment materials as a key factor in restoring implant-supported single crowns. Thus, the null hypothesis that different materials of crown and customized abutment would not result in different stress distribution pattern and values in hybrid customized abutment complex was rejected.

In the current study, the analysis of von Mises stress distribution was conducted on models restored with zirconia crowns (Fig. 3), because these models exhibited the highest average stress in customized abutments compared to those using different crown materials, as indicated in Table 3. Furthermore, zirconia is frequently selected for posterior restorations due to its durability and mechanical properties, making it a popular choice among dental materials [32]. The von Mises stress distribution analysis showed consistent patterns across models, with stress concentrations at the Tibase–abutment interface, particularly in models utilizing low-stiffness abutment materials such as PEEK variants (Fig. 3E, F and G). This result aligns with findings from Kaleli et al. and Deste Gökay et al., who reported that low-modulus abutment materials tend to shift stress to the implant connection area, potentially increasing the risk of mechanical complications [8, 13, 19].

The analysis of peak, volume average von Mises stress and peak (in magnitude) principal stresses demonstrated that customized abutment materials significantly affect stress levels in testing models more than crown materials, as illustrated in Tables 2, 3, 4 and 5. To facilitate discussion, the authors have highlighted key findings for each component:

Considering crown component, it was observed abutments with a lower Young’s modulus exhibited greater volume average stress, especially when coupled with high Young’s modulus crown materials like zirconia. Crowns on PEEK abutments displayed approximately three times the volume average stress higher compared to those crowns on zirconia abutments. This difference was attributed to the varying material properties, excessive differences in material properties led to stress shielding effect [19, 33], which hindered effective stress transfer from the crown to the abutment and other components. The data indicated that peak stress values increased when low Young’s modulus crown materials were coupled with high Young’s modulus abutments. However, when lower Young’s modulus crowns were placed on lower Young’s modulus abutments, the peak stress decreased. This suggests that matching the mechanical properties of crowns and abutments can mitigate stress concentrations in implant-supported restorations [33]. This stress-shielding phenomenon has also been reported by Epifania et al. and Ausiello et al., emphasizing the need to match mechanical properties between components for optimal load transfer [18, 20]. Consequently, using low Young’s modulus crown on high Young’s modulus abutments was found to increase stress absorption due to higher stress concentrations in the crown materials. These crowns of PEEK-variants exhibited ductile behavior, resembling polymer-like materials, which significantly affected their mechanical response under load [7, 33].

Considering the customized abutment components, it was observed that the material properties of the abutment significantly influenced stress levels more than crown materials, as demonstrated by the dominant changes in peak and volume average von Mises stress values based on abutment materials shown in Table 3 and stress concentration are in Fig. 5. The high Young’s modulus material group (Zir, Ti, LDS, and PICN) exhibited similar peak von Mises stress values around 86–96 MPa, whereas the PEEK-variants showed lower peak stress values approximately 41–74 MPa. Despite this, all abutment materials displayed stress levels well below their yield strengths [34]. Additionally, high Young’s modulus abutment was associated with higher volume average stress values, likely due to the stiffness of materials to better withstand compressive loads distributed from the crown, as observed in Zir, Ti, LDS, and PICN, which showed the peak values von Mises stress, minimum principal stress and volume averages von Mises stress due to their minimal deformation behavior. From a mechanical perspective, abutments serve as the primary mediators for occlusal load transfer [35]. Based on the results, abutment materials with high Young’s modulus, such as Zir, Ti, LDS, and PICN, demonstrate superior load transfer capabilities compared to those made from materials with PEEK-variants. Our findings are consistent with those of Sellan et al., who highlighted that materials with greater stiffness better resist functional forces and contribute to long-term structural stability in implant restorations [36]. This indicates that choosing abutments with high Young’s modulus can significantly enhance the effectiveness of load distribution in hybrid customized abutment complex.

Tibase serves as a crucial connector between the customized abutment and the implant fixture through the abutment connector [8]. The volume averages von Mises stress results indicated that the titanium base abutment had the highest accumulated volume average stress than others components. Volume average stress ranged from 70 to 99 MPa for materials from the PICN and PEEK-variants groups, whereas Zir, Ti, and LDS exhibited stress ranges from 55 to 59 MPa. Notably, peak minimum principal stresses were significantly higher in models with PEEK abutment, with values approximately twice as high as those in other models. Additionally, principal stress distribution (Fig. 6) illustrates that titanium abutments in models restored with PEEK-variant customized abutments exhibit large areas of high (in magnitude) principal stress concentration at the implant-abutment connection. Furthermore, a high stress concentration zone was observed at the retention post of the Tibase in models restored with PEEK abutments (Fig. 6G and P). When considering the peak principal stress values (in magnitude), it was observed that Tibase in models with abutment materials possessing a low Young’s modulus, such as 10.5 GPa for PEEK-G and 3.5 GPa for PEEK, exhibited higher peaks in the magnitude of minimum principal stresses, reaching − 400 and − 550 MPa respectively. In contrast, other materials typically displayed peak principal stresses around 300 MPa and − 300 MPa. In clinical settings, the choice of customized abutment materials significantly influenced the mechanics of the Tibase. It is recommended to use materials with a high Young’s modulus to improve load transfer and reduce high stress concentrations.

When considering the results, it was shown that the mechanical behavior of the hybrid customized abutment complex was related to compression behavior. The highest loads were received by the Tibase, which displayed highest volume average von Mises stress values between 50 and 90 MPa, depending on the materials of the customized abutment (and crowns?). This was followed by the abutment screw at 44 MPa, the implant at 20 MPa, the customized abutment at 3–9 MPa, and finally the crown at 0.7-6 MPa. This sequence indicates that the load was transferred from top to bottom, with the Tibase playing a crucial role in the functionality of this complex. Therefore, materials for the hybrid customized abutment complex selection should aim to reduce stress concentration mismatches and prevent mechanical complications. Based on the findings of this study, the use of high Young’s modulus materials (such as Zir, Ti, and LDS) for abutments could increase the stiffness in the system of the hybrid customized abutment complex. This increase in stiffness may enhance the homogenous transfer of loads from top to bottom. In clinical situations, the selection of materials for restoring the hybrid customized abutment complex should consider the mechanical aspects of load transfer in addition to aesthetic and biological responses.

In this study, the control models represented the commonly used equal crestal placement protocol for dental implants, while the testing models represented implants placed in a 1.5 mm subcrestal position. Results indicated that the subcrestal models exhibited higher peak and volume average stresses, especially in the Tibase, compared to the equal crestal placement [9]. Although peak stresses increased, they did not exceed the material limits. This increase in stress can be attributed to the deeper placement of the implant, which lengthens the lever arm of the abutment and crown, resulting in increased mechanical loads on the implant system. Clinically, the 1.5 mm subcrestal placement is unlikely to induce mechanical problems, though scenarios with crown height space exceeding 15 mm may require additional consideration [37].

The limitations of this study stem from its in-silico nature, underscoring the necessity for clinical validation to align the simulation outcomes with actual clinical scenarios and material properties. Accurately modeling the shape of implants and their components, especially customized and titanium base abutments, differs from real-life applications in each patient. The results of this study allow for comparisons between different implant designs, notably those featuring internal hexagons and Morse taper abutment connections, which impact mechanical behavior. Insights gained could guide the selection of optimal materials for hybrid customized abutment complexes and aid in predicting potential complications.

## Conclusions

Based on the findings from this 3D nonlinear contact analysis FEA study, several conclusions were drawn:

1. The selection of crown and abutment materials significantly influences stress distribution and concentration within hybrid customized abutment complexes. Among these, abutment materials demonstrated a greater impact on mechanical outcomes compared to crown materials.
2. Abutments fabricated from materials with a high Young’s modulus contributed to increased stiffness in the system and showed more favorable stress distribution characteristics in the titanium base component.

These conclusions reflect trends observed in a simulated environment. As this study was conducted exclusively through computational modeling, further in vitro and clinical investigations are necessary to confirm the mechanical performance and validate potential clinical relevance.

## Data Availability

The data that support the findings of this study are not openly available and are available from the corresponding author upon reasonable request.
